# Transgender and gender diverse autistic adults’ experiences of (un)belonging

**DOI:** 10.1371/journal.pone.0338569

**Published:** 2025-12-18

**Authors:** Katie Munday, Steven K. Kapp, Charlotte Morris

**Affiliations:** 1 School of Psychology, Sport and Health Sciences, University of Portsmouth, King Henry Building, Portsmouth, United Kingdom; 2 School of Area Studies, Sociology, History, Politics and Literature, St.George’s Building, University House, Portsmouth, United Kingdom; Nagoya University: Nagoya Daigaku, JAPAN

## Abstract

**Introduction:**

Clinical impressions suggest a significant overlap of autistic as well as transgender and gender diverse identities, implying a need for research that explores TGD autistic experiences in greater depth, including experiences of (un)belonging.

**Methods:**

We shared trans and gender diverse autistic adults’ experiences of belonging and unbelonging to contribute to knowledge around their lived experiences. We present findings from biographical narrative interpretive interviews with thirteen trans and gender diverse autistic people (aged 20–50). We used reflexive thematic analysis to generate themes across three levels of belonging: macro, meso and micro. Analysis within these levels clarified (un)belonging within power dynamics and structures, as well as collective and individual identities.

**Results:**

Participants discussed their experiences of (un)belonging across three levels: macro, encompassing work and volunteering; meso, including education, gender identity healthcare, and neurodivergent groups and spaces; and micro, including relationships and creating chosen families. Participants faced workplace exclusion and healthcare gatekeeping, often turning to self-employment or community spaces for inclusion. Some found belonging in autistic or queer communities, while others struggled with accessibility and sensory barriers.

**Conclusion:**

Chosen families and communal living were key sources of affirmation and support for participants, highlighting how belonging for TGD autistic people can be shaped through intersecting structural, social, and interpersonal factors.

## Introduction

Awareness and visibility of trans and gender diverse individuals and identities, referred to as “the transgender tipping point” [1], has grown significantly in recent years. This shift has been accompanied by a rise in academic and personal literature aimed at enhancing visibility, fostering inclusion, and creating a sense of belonging within broader society for transgender and gender diverse (TGD) individuals across the global North [2–4]. Increased visibility, however, has not necessarily translated into structural equality or safety for transgender people. While media representation and policy advocacy have expanded, systemic issues such as healthcare discrimination, employment inequality, and violence against TGD individuals persist at alarming rates [5]. Many transgender and gender diverse adults continue to face discrimination and unbelonging in the workplace and labour market [6], as well as ongoing systemic and interpersonal barriers within healthcare [7]. This discrimination often includes implicit transphobia, such as misgendering and being overlooked for promotion [8], and explicit transphobia, involving harassment, abuse, and physical violence [9]. Due to the permeation of these experiences across different life spheres, transgender and gender diverse people are at heightened risk of homelessness [10], and domestic violence [11].

Similarly, awareness and understanding of autism have expanded significantly over the past two decades, marking a shift from deficit-based medical models toward neurodiversity and inclusion in the global North [12]. Despite this shift, autistic adults continue to experience discrimination and unbelonging across different levels, including health and social care [13–16]. In a survey of 209 autistic adults and 228 non-autistic adults in the U.S, Nicolaidis and colleagues [15] found that autistic participants had significantly greater unmet healthcare needs and lower satisfaction with patient–provider communication. Miscommunication is also a barrier to autistic adults gaining and maintaining employment and higher education [17,18]. These miscommunications can be explained by the Double Empathy Problem [19] in which misunderstandings—and the associated blame—occur *between* individuals with differing neurotypes. This effect is perhaps more severe for TGD autistic individuals [50] as the power imbalance in healthcare typically favours the (typically) more educated and privileged practitioners, placing marginalised individuals in a vulnerable position when seeking care. The issue of patient blame within healthcare is deeply ingrained and can include misogyny, transphobia, ableism, and ageism; the double empathy issue is profoundly intersectional [19].

While awareness of transgender and autistic identities has increased in recent years, both groups continue to experience marginalisation, discrimination, and exclusion [6–8,13–18]. TGD autistic adults sit at the intersections of these (un)belongings. Feelings of unbelonging are multiplied, creating complex experiences of unbelonging across social and institutional structures, including systems that govern access to housing, travel, employment, and other essential structures [20–22]. Previous research has tended to emphasise prevalence rates rather than lived experience, establishing that transgender identities are significantly more common in autistic populations than in non-autistic populations [23–25]. There is also a growing area of research which focuses on how to support TGD autistic individuals in healthcare settings, with specific attention to gender identity healthcare [26–29]. However, there remains little exploration of TGD autistic people’s experiences of belonging in daily life or how structures such as work, education, and healthcare shape that belonging. Our work begins to address that gap through centring TGD autistic narratives.

### Conceptual framework

Belonging has been defined as a unique and subjective experience that relates to a desire for interpersonal connection related to feeling included, appreciated, welcomed, and understood [30]. The key characteristics of belonging include autonomy, choice, and commitment and can be associated with objects, experiences, events, places, and land, as well as with other people [31]. Crucially, one’s commitment to relationships and locations depends on the value one assigns to them. Conversely, unbelonging happens through devaluation, exclusion, and invalidation. Places and temporalities, intertwining with personal narrative, power dynamics and lived experiences, can foster (un)belonging [32,33].

In the current study, we applied this understanding of (un)belonging across a three-level framework – macro, micro and meso. The macro level included abstract communities such as nationality and religion. The meso level incorporated association with collective organisations such as political parties and social movements. Finally, the micro level was situated in interpersonal interactions with friends, family and local community [34]. Analysing within these levels elucidated the connections between political and social structures and the creation of collective and individual identities. We appreciated that there were relational, spatial, and cultural dimensions of (un)belonging that were inextricably linked [32]. These levels have been utilised in various studies to gain a deeper understanding of (un)belonging, particularly among migrants and immigrants [35,36].

We applied a biographical narrative interpretive method of investigation [37] to explore the experiences of TGD autistic adults, aiming to understand the interconnected nature of life stories shaped by personal perceptions, contexts, and events [38]. Utilising a narrative approach that focused on the voices of TGD autistic people allowed us to capture rich and intricate experiences [39–41]. We believe the act of storytelling (both in telling and performing) is central to our humanity and that the narratives we share about ourselves blend our self-identity and sense of belonging within broader societal contexts [40]. Storytelling transcends mere creativity; it serves a political purpose by giving individuals agency to interpret and share their experiences while asserting their role in the world [42]. The narratives in this study add to existing research on belonging and accessibility within healthcare, [14,26,27,43] education [44], and the workplace [45], as well as broader lived experiences of TGD autistic individuals [37–40]. This research is the first to apply a biographical narrative interpretive method of investigation [37] to investigate the experiences of TGD autistic adults.

## Methods

K.M. applied the Biographic Narrative Interpretive Method [37] during eight, one-time, hour long, online video conference interviews. K.M., with support from C.M., carefully constructed a single question to begin the narrative portion of the interview process. K.M. then developed subsequent questions from the topics raised. Positioning the ‘whole story’ as the object of study, as opposed to essentialising participants to their various identities, gave participants agency over their stories [38].

K.M. also collected data through five email interviews, created in response to prospective participants’ needs. The written interviews used a mix of closed and open-ended questions. These interviews included pre-written questions, with examples and maximum word counts (ranging from 200 to 500 words, depending on the question). The email interviews did not follow the Biographic Narrative Interpretive Method, as this would not have been appropriate [37]. These two modalities had the potential to yield different yet complementary data regarding spontaneity, depth, and narrative structure. Due to this, the first round of analysis, specifically reading the data, generating initial codes, and searching for themes, was done separately. Once K.M. and C.M. were satisfied that reflexive thematic analysis [46] would be appropriate for drawing together the stories from both modalities; the other stages of analysis were completed.

Participants provided demographic information through close-ended questions on age, pronouns, and ethnicity. The questions on ethnicity, gender, and pronouns included free-text boxes to avoid othering already marginalised groups and accommodate those with multiple identities [23].

Study information was distributed to participants three days before their interviews, as autistic individuals often require more time to process new information [47]. This allowed participants to process the information and raise any questions or concerns before the start of their interview, in line with ethical and inclusive practice. Due to the nature of the topics discussed in this study, participants received a list of support resources within the participation information sheet and the debrief sheet. They were also able to have a support person (friend, family member or personal assistant) present at their interview. However, no participants opted to have a support person present.

Transcripts were recorded through audio within video interviews and corrected by K.M post-interview. Participants were sent their transcripts post-interview for comment or correction. K.M. used their previous training from their bachelor’s and MRes courses to support the methodology. K.M kept field notes throughout the interview process to reflect on their own understandings and experiences.

### Participants and recruitment

This study represents an in-depth examination of the individual narratives of thirteen trans and gender-diverse autistic adults, focused on adults aged 20–50, to account for the late diagnosis of autism [48,49] and ‘later life’ transitions [50]. K.M. collected data from thirteen participants who were autistic (either formally or self-diagnosed) and TGD adults who could complete an interview in English (see Table 1). Recruiting self-diagnosed participants allowed for a more diverse participant pool, as an autism diagnosis can be unwanted or inaccessible [51]. Non-clinical recruitment also allowed for more diverse participants and narratives, appreciating that not all gender diverse people seek medical intervention [52]. The focus was on trans-masculine, trans-feminine and non-binary participants (although many participants held several other gender identities, such as agender). Following the range of identities recruited in previous studies allowed us to contribute to this emergent area [23,53,54].

**Table 1:** Participant demographics.

*Pseudonym*	*Location*	*Age*	*Ethnicity*	*Gender*	*Pronouns*	*Interview modality*
Sam	UK	27	White	Agender non-binary	They/them	Video conference
Jay	US	31	White	Agender non-binary	They/them	Video conference
Derek	UK	20	White	Trans man/man	He/him	Video conference
Scott	S. Africa	31	Mixed	Trans man/male	He/him	Video conference
Micky	US	25	White	Transmasculine non-binary	They/them	Video conference
Kim	UK	50	White	Non-binary	She/her	Video conference
Robin	US	26	White	Non-binary transmasculine	They/him	Video conference
Toby	UK	33	White	Non-binary	They/them	Video conference
David	US	21	White	Transgender man	He/him	Written
Anthony	Australia	33	White	Trans male	He/him	Written
Greg	US	25	White	Trans man	He/him	Written
Anette	France	27	White	Non-binary, queer, agender	Elle/ she/they	Written
Ronnie	China	34	Chinese	Non-binary	They/them	Written

K.M. recruited participants between November 1st, 2021, and April 30th, 2022, through an advert on private social media groups for autistic people who are lesbian, gay, bisexual and/or transgender+ (LGBT+). K.M. had been an active member of these groups for several years and used their affiliation to gain a convenience sample [55]. K.M did not have direct contact with participants before recruitment. Due to the nature of these groups, participants were recruited from across the world (See Table 1).

Participants provided written informed consent. No participants refused to participate or dropped out. All participant data was anonymised, stored and shared in line with the Data Protection Act, 2018 [56]. The University of Portsmouth Faculty of Health and Social Sciences Ethics Committee provided ethical approval for this study. The procedures used in this study adhere to the tenets of the Declaration of Helsinki.

The autism community (including autistic adults) have reported opposition to openly sharing data related to concerns about the misuse of data, harmful narratives arising from secondary researchers’ interpretations, and the risk of identification (for example, being forced to disclose their autism diagnosis or identity, and backlash from employers, peers, and the police. [57,58] These ethical risks are amplified as participants’ autism and gender identity are not necessarily public. This is further complexified by the current cultural contexts of many participants (e.g., in the U.S. and the U.K.) where transgender people’s gender identity is no longer legally protected, further legitimising concerns about employer or police retribution [59]. We take a position in line with leading open research scholars against mandatory open data sharing for qualitative research, which is especially important for vulnerable populations with sensitive data [60,61].

### Analysis

K.M. applied experiential reflexive thematic analysis [46] through NVivo to identify variations and commonalities across narratives using a six-step approach: reading the data, generating initial codes, searching for themes, revising themes, labelling and defining themes, and producing an overall report [40,60]. K.M. created codes using an open-coding approach, in which they identified important themes and patterns from the narratives based on their personal experiences, while focusing on the participants’ lived experiences, perspectives and needs. The analysis progressed from a description of the data to an interpretation of the themes, informed by wider literature, to a discussion of their implications across three levels of (un)belonging [34]. K.M. and C.M. engaged in iterative discussions to ensure the themes were grounded in the data [40]. Together, they reviewed and further developed the codes, subthemes and themes to ensure clarity and avoid repetition [57]. C.M. maintained oversight of the analysis to ensure the process remained rigorous and systematic. Due to the nature of reflexive thematic analysis, in which each story/data is not considered to contribute to a saturation point [40], we recruited a number that suited the timescale of K.M.’s MRes course.

### Reflexivity

K.M., the principal investigator, is a trans-masc non-binary autistic person who was studying for their MRes (Master of Research) at the time of data collection. C.M., the direct supervisor, is a cisgender neurodivergent scholar with expertise in Gender Studies and narrative methodology. S.K.K., current supervisor (Doctor of Philosophy), is a cisgender autistic autism researcher. K.M. reflected on their own experiences, in line with reflective thematic analysis, which considers attending to subjectivity a key feature of analysing data [46,62]. The participants were made aware of K.M.’s position and identity before their interviews.

### Findings

Participants shared their experiences of (un)belonging at all three levels. They discussed their experiences of (un)belonging in their work and volunteering, highlighting challenges in accessing preferred employment, transitioning between jobs, and resigning from positions. Even though many participants lacked necessary adjustments, a significant number were engaged in education or employment, with some being self-employed (like Greg and Anthony). Additionally, participants elaborated on their experiences of (un)belonging within educational settings, queer spaces, and neurodivergent communities. They also shared their experiences of gatekeeping and unbelonging in medical and mental health services, including insufficient professional knowledge, limited funding or insurance coverage, and overt transphobia and ableism. Participants also discussed their feelings of (un)belonging in their relationships and family dynamics, which included queer platonic relationships and chosen families, often formed as an alternative to their biological families (see Table 2 for representative quotes). K.M. has presented the additional subtheme of participants’ suggestions for TGD autistic research elsewhere [22].

**Table 2:** Representative quotes.

Theme 1: Macro level of belonging	Representative Quotes
Theme 1.1: Work and Volunteering	•“I have only mentioned my autism in passing at work because while it is not explicit, I get the feeling that there is too much ableism going around. My boss is great, and I love being there, but I have watched them make fun of [Neurodivergent] people. I also know a co-worker is a Trump supporter and while she has been nothing but nice to me, it makes me feel unsafe to fully come out.” •“I did tell [my university tutor] that I was autistic at the point that I got my diagnosis, and she was like “Oh, this is an interesting career choice for an autistic person.” And that was the start of a whole avalanche of different kinds of ableism.” •“I’m out as trans at work, but I’m not out as autistic […] because autism is less visible and when it is visible, it is far less informed and there’s far less incentive for people to be informed about it right now […] As a transgender person, there’s more institutional support but presently as a neurodivergent person, it is highly dependent on your manager.” •“I have a lot of sensory issues. A lot of workplaces are difficult for me because of things like fluorescent lighting, too much noise other people in the workplace or patrons using strong perfumes or deodorants. I have struggled very much in the employment market, as the jobs I’m most qualified for seem to be the ones that are least suited to me in terms of sensory needs.” •“I trained as a classroom teacher in my twenties, and I went for loads of interviews, I never got past an interview, they always picked someone else […] It just didn’t work out being a classroom teacher, but it worked out for the best because I’m much happier teaching one-to-one.”
**Theme 2: The meso level of belonging**	**Representative Quotes**
Theme 2.1: Education and training	•“I enjoy learning and studying and enjoyed using it to live somewhere else for three years […] it would be just like a nice decent excuse to live in [Ireland] without also losing all my benefits and medications. And have a reason and a purpose to be there rather than just turning up.” •“I want to go to grad school and get an adult MSW, MPH degree and use that to do some community organising” •“Work is much harder. Without a diagnosis, I don’t say anything about autism and often end up with shutdown at the end of the day.” •“Some of them [my students] know I’m gay but I’m not out as Autistic, because I don’t know how that would go down. Not quite sure if it would go down very well and I don’t require any additional support.”
Theme 2.2: Gender identity healthcare	•“I’m autistic and I’m in a building with literally thousands of other people […] I’m getting overstimulated, I’m getting overwhelmed, my anxiety is shooting through the roof.” •“Hospitals are very sensory-overwhelming places […] being in a hospital for even a day feels like I may as well be climbing a mountain.” •“Whilst going through any medical or therapeutic sessions as a diagnosed Autistic person sometimes it can take longer. I’ve read that and I’ve had people who are formally diagnosed say ‘yeah, that’s correct, it has taken me longer to be able to access the correct healthcare and go through everything’ - that their process takes longer because they are Neurodivergent.” •“A potential issue with getting top surgery is that hospitals are very sensory-overwhelming places and that has made me put off even looking into the process. I do not do phone calls well, and so figuring everything out with insurance and surgeons, and then being in a hospital for even a day feels like I may as well be climbing a mountain. It’s a bit much for me to handle by myself, to say the least.”
Theme 2.3: Neurodivergent groups and spaces	•“People I have fixations on do tend to be neurodivergent which happened even before I had my diagnosis where there would just be one [autistic] person in the room that I would be very interested in immediately […] I just kind of recognise that sameness and am quite drawn to that.” •“I am in social media groups for both autistic and trans people. It’s great that I can find a community of people like me and know that I’m not alone.” •“So, whilst I really enjoyed going out and dancing with my friends and maybe getting a bit drunk. I used to get so drunk because I was in physical pain from my chronic conditions, but also from like the sensory ‘bleurgh’ awfulness that is just going out clubbing as an Autistic […] I used to smoke as well for ten years I smoked because it was like my crutch of how to have, like a socially acceptable ‘out’ of a sensory break […] it’s socially acceptable to get really f**king drunk, or smoke a lot, and go out for fag breaks all the time, instead of being like ‘Hey, I need a sensory break - this is too much.” •“I’ve met quite a few friends at an autistic conference I’ve been to 3 times in person and 2 times online. I’ve made quite a few friends there. I’m also in a theatre group which is for autistic people and others […] I’ve been involved in that for three years now.”
**Theme 3: The micro level of belonging**	**Representative Quotes**
Theme 3.1: Relationships and social connections	•“My favourite hobbies are things I can do by myself so when it became a decision between my pastimes or hanging out with people, I preferred to stay home.” •“I’ve always had different interests from the other kids, currently, I’m really into taxidermy and bones and stuff like that […] People wanted to play kiss chase in the playground, and I was climbing trees and finding books and stuff, and being in my own company, and I was happy in my own company.” •“I have a wide range of hobbies. Mostly ones that I can practice by myself, like astrophysics, drawing, music, or arts. […] I like to go to concerts and museums, but often by myself. I find it hard to cope with what I see and who I am with at the same time.” •“I struggle socially in any context that isn’t one-to-one […] because I do experience situational mutism in groups it’s quite humiliating. I even struggled to go to autistic groups, because of having had so many negative experiences in groups where I’ve just not been able to talk. So, even though I’m aware that it could be quite different amongst autistic people, I still have that kind of reticence.”
Theme 3.2: Creating Chosen Family	•“Not having a typical family structure is something I want to work towards because I’m constantly having to accept that I don’t meet societal standards in basically all the ways possible I could not meet them. Friends are the centre of my relational world in a lot of ways and my family don’t necessarily get that or recognise it or understand that.” •“I’d quite like to have kids. It’s something that I had to think about before medically transitioning due to the fertility issues that come along with testosterone. I don’t want biological kids […] but if it came around that there was a kid that needed a home, I’d be perfectly happy to take them in.” •“I find like, with like relationship anarchy stuff and polyamorous descriptors quite helpful things like describing friends as anchor partners because I do consider one friend a life partner even though it’s a platonic relationship […] I’ve got polyamorous friends where some of the language they use really resonates with me, it’s definitely something that makes a lot of sense to me, as a way of existing with other people.” •“I would like a commune, for all of my sick, queer and Disabled friends […] My friends are all over the country all over the world, all dealing with very similar issues to me, whether it’s health care issues, or funding or living space or care, and work and stuff like that it just feels and sounds much better if we could all just move in together and help each other doing the things that someone else can’t do but you can do.”

## Theme 1: Macro level of belonging

### 1.1 Work and Volunteering

All participants highlighted issues with gaining support at work. Challenging environments at work, both social, emotional, and physical, were barriers to belonging. These challenges persisted whether participants chose to disclose their TGD and autistic identities or not. Anette shared that they didn’t tell others about their diagnosis and would “often end up with shut down [sic] at the end of the day.” Anette explained how she experienced shutdowns – an extreme case of inertia – whilst at work due to being consistently overwhelmed. Anette felt that their needs would not be supported by their colleagues at work due to their not being formally diagnosed as autistic. Similarly, Ronnie shared, “I have only mentioned my autism in passing at work because […] I get the feeling that there is too much ableism going around”. Ronnie had mentioned to some work colleagues that they were autistic; however, they were uncomfortable coming out as non-binary due to the political opinions of other staff (“I also know a co-worker is a Trump supporter”).

Toby experienced ableism from their university tutor, who suggested that counselling was “an interesting choice for an autistic person”. This conversation started an “avalanche of different kinds of ableism”. Toby felt frustrated and confused that someone with a counselling background would be “so explicitly judgmental and ableist”. Similar issues prevented Micky from being openly autistic at work (“Autism is less visible and when it is visible, [people] are less informed”). Micky articulated how they felt much more confident and protected in their belonging as a transgender person in their workplace. They said that working at a large corporation, with over 5,000 staff members, made the processes for transgender inclusion (such as changing names and pronouns on work documents) easier for the company to manage. He suggested that there was no all-encompassing way to support autistic people at work.

Toby shared that they struggled to find and keep employment: “I tend to work in two-year increments in a particular place. I get really burnt out every couple of years and discontent with the work culture.” Toby described a persistent dissatisfaction with workplaces and colleagues, leaving them to change jobs frequently. Jay shared similar experiences of unbelonging in workplaces due to unaccommodated sensory and social needs relating to “fluorescent lighting, too much noise and other people in the workplace using strong perfumes or deodorants”. They often had unmet sensory needs, including the use of fluorescent lighting and staff member perfumes triggering migraines, both of which were ignored or used intentionally to berate them at work. This is an important factor for autistic people as they often experience atypical sensory sensitivity, which can be either hypo, hyper or a mix of sensitivities [63].

Participants often felt that they were missing job opportunities due to the unaccommodating nature of potential workplaces. Unfortunately, many employers lack knowledge of autistic experiences and therefore do not adjust the working environment or expectations for autistic workers [64]. This, coupled with workplace bullying and prejudice, use of overly complex bureaucratic processes, inflexibility in work hours and lack of clear communication, leaves many autistic people unemployed or underemployed in job roles which do not match their capabilities [64,65]. David and Kim found it difficult to get a job or create a career in the way they had hoped; Kim became self-employed to better manage her work environment, and David aspired to be a flight attendant despite reflecting that he lacked “some social skills that could be important.” Unfortunately, in the UK, just 22% of autistic people aged 16–64 are in employment, which is amongst the lowest employment rates amongst disabled people in the UK [66]. Unemployment rates are similar for transgender people in the USA, as they are twice as likely to be unemployed and make 32% less money per year than their cisgender counterparts [67]. Despite higher rates of transgender identities in the autistic community [23,24] there remains a lack of research on employment rates and employment experiences of TGD autistic adults.

## Theme 2: The meso level of belonging

### 2.1 Education and training

Some participants had gone back to college and university to retrain for work “I’m currently retraining in the hope of finding more appropriate work” (Anthony) and “I want to go to grad school and get an adult MSW, MPH degree and use that to do some community organising” (Micky). There has been a steady global increase in autistic adults accessing college and university [68] using their passionate interests, attention to detail, memory skills, and desire to learn to their advantage [69]. Sam shared, “I enjoy learning and studying and I enjoyed using it to live somewhere else for three years”. Anette, Micky, and Sam planned to undertake qualifications to improve their job prospects. Sam saw university as a way of making friends as well as increasing their knowledge and work-based skills. However, they were concerned about transferring their disability benefits and medical needs to another country.

### 2.2 Gender identity healthcare

Various participants spoke about their gender identity healthcare goals, including “top surgery” (Anthony and Greg) and “transitioning almost fully except for bottom surgery” (David). Anthony shared that he experienced gatekeeping and unbelonging in gender identity health care due to his diagnosis of autism. The continued pathologisation of autistic people can cause many TGD autistic adults to disengage entirely from gender identity healthcare [43]. Anthony and Micky experienced healthcare providers who treated them “as children” as soon as they were made aware that they were autistic.

Healthcare practitioners frequently lack sufficient training regarding the experiences of autistic individuals and those who are transgender or gender diverse, impacting the suitability and availability of care. Practitioners may understand autistic or TGD healthcare needs, but they seldom recognise how these categories can intersect to form unique healthcare needs [20]. Participants shared that some practitioners understood the participants autistic needs but were unwilling to help with their gender identity needs: “There is so much gatekeeping when it comes to being officially diagnosed as autistic, and medically transitioning, and I’ve found that my psych for one doesn’t feel comfortable assisting with the other” (Anthony). This meant Anthony had to finance additional sessions at “several hundred dollars” with psychiatrists to get the care they needed. Greg also experienced additional costs to their healthcare and explained that they were unlikely to access gender identity healthcare: “top surgery is too expensive”. Participants living in the U.S. shared their struggles with administration and bureaucracy within privatised healthcare.

Some participants also felt unbelonging due to sensory and social overwhelm within medical settings, including Scott, who explained that gender identity healthcare settings were too busy and sensorially overwhelming. He described the settings in which he could find help as places which added to his overwhelm: “I’m autistic and I’m in a building with literally thousands of other people. I’m getting overstimulated, I’m getting overwhelmed, my anxiety is shooting through the roof”. Greg shared a similar experience: “Hospitals are very sensory-overwhelming places; being in a hospital for even a day feels like I may as well be climbing a mountain”. Navigating gender identity healthcare is demanding; patients need to organise appointments, admin and correspondence whilst experiencing a highly emotional personal process [29]. Greg explained that he would need significant help from hospital staff to be able to access gender identity healthcare. Greg and Scott both experience unbelonging at hospitals due to sensory violence despite living over 8,000 miles apart. Some participants gave recommendations to improve access and belonging for gender identity healthcare. Scott suggested that healthcare practitioners should “understand how things intertwine and how to interact with neurodivergent people”. Similarly, Toby shared that they just wanted to be “listened to and taken seriously and have my gender identity recognised”.

### 2.3 Neurodivergent groups and spaces

Most participants shared that they enjoyed friendships with other autistic people to whom they were often drawn: “I am fortunate to have an excellent group of friends […] it turns out we’re all neurodivergent” (Anthony) and “A lot of my friends are autistic or otherwise neurodivergent” (Robin). Toby shared, “People I have fixations on tend to be neurodivergent, which happened even before I had my diagnosis.” Anthony, Toby and Robin explained how they were drawn to other neurodivergent people, and they described an intrinsic ability to recognise similar others and feel belonging with them. Autistic adults have been found to interact with other autistic people more readily than they do non-autistic people. Seeking out others with whom they share identity, and culture makes it easier for many of them to interact in ways that support their needs and interests [70]. Belonging, related to an emotive feeling of recognising yourself in others, can enable autistic people to develop a better sense of self-worth [71].

Kim shared how she made connections at a yearly autistic learning and social space, which she went to regularly. She also shared that she was part of a theatre group for autistic people and that “Before that, I can’t say I did have many friends, especially when I was a younger person, I didn’t have any friends at all.” Kim has created relationships and connections with other autistic people, including her wife (“my wife is also non-binary and autistic!”) and attends autistic groups and conferences. She described having issues with establishing and sustaining relationships before she was diagnosed in her early forties. Kim now belongs to autistic-only groups and spaces, making it easier for her to connect and belong with others. Ronnie had similar experiences: “I am in social media groups for both autistic and trans people. It’s great that I can find a community of people like me and know that I’m not alone.”

Kim and Ronnie both shared that they had few friends before joining online and in-person autistic groups. They explained how they felt a sense of belonging within LGBT+ autistic spaces, including those at autistic conferences: “The LGBT+ meeting was just like a support group, it felt amazing that there were so many of us in that room”. However, not all LGBT+ spaces are autistic-friendly, as Sam shared, “I used to get so drunk because I was in physical pain from my chronic conditions, but also from the sensory ‘bleurgh’ awfulness that was just going out clubbing as an autistic.” Sam explained how they felt unbelonging in LGBT+ nightclubs as their sensory sensitivities were not supported. They suggested that it was more socially acceptable to be overly intoxicated than to ask for a sensory break.

## Theme 3: The micro level of belonging

### 3.1 Relationships and social connections

All participants spoke of experiencing social anxiety, especially when meeting new people, as well as difficulties in creating and maintaining new relationships: “I don’t have a great history of keeping friends” (Greg). Some participants shared that they preferred to follow their interests and hobbies rather than connect with others; “My favourite hobbies are things I can do by myself”, “I’ve always had different interests from the other kids […] I was happy in my own company” (Derek) and “I like to go to concerts and museums, but often by myself. I find it hard to cope with what I see and who I am with at the same time” (Anette). Derek, Greg, and Anette preferred to socialise one-on-one, online or around their specific interests, which have been found to improve autistic adults’ wellbeing and satisfaction [72].

Toby shared why they preferred one-to-one social situations: “I struggle socially in any context that isn’t one-to-one […] because I do experience situational mutism in groups, it’s quite humiliating”. Toby was so anxious about their situational mutism that they had yet to try autistic spaces, “Even though I’m aware that it could be quite different amongst autistic people, I still have that kind of reticence”. Despite high levels of social anxiety among participants, many spoke about the great love and belonging they felt in their close relationships, especially those they had with other autistic people (Kim, Anthony, Toby and Derek). Autistic people often thrive in relationships where their unique ways of expressing themselves are acknowledged and valued [71,73].

### 3.2 Creating Chosen Family

Most participants were happily living in non-traditional households or family structures, including living alone, living with animals or living with a partner and their “fur baby” (their cat). Trans and queer people frequently establish family connections outside of their biological families, often as a result of estrangement following their coming out or because they do not conform to traditional concepts of the ‘nuclear family’ [74]. Communal living and creating a chosen family were an aspiration of Derek, Micky and Toby. Toby spoke about their understanding of family: “Not having a typical family structure is something I want to work towards […] Friends are the centre of my relational world in a lot of ways.” They shared that they had started to create their own chosen family with their queer platonic “life partner” but wished that others, including their biological family, would appreciate the importance of these connections. Toby had a keen sense of belonging with their friends and believed that their experience of friendship was different from that of most people.

Derek showed an interest in adopting or fostering children: “I’d quite like to have kids. It’s something that I had to think about before medically transitioning due to the fertility issues that come along with testosterone”. Derek spoke about the choices of fertility provisions, including whether to freeze his eggs, due to infertility issues which can occur while taking hormones. He explained that he may adopt or foster children in the future, but he did not want to carry or birth a child. Micky aspired to have a chosen family: “When I’m older, I would like to be able to foster and adopt kids.” Fertility preservation is an important choice for transgender and non-binary individuals undergoing hormone replacement therapy and/or gender-affirming surgeries. At present, there is limited information regarding the effects of testosterone therapy on future fertility, leading to the absence of a well-defined optimal period for pursuing fertility preservation [75]. Additionally, research on live birth rates associated with preserved eggs is scarce, and the procedure for egg retrieval is invasive, potentially leading to emotional distress for patients [75,76].

Sam described the challenges faced by themselves and their friends simultaneously throughout the UK and beyond (“My friends are all over the world, are dealing with very similar issues to me, whether it’s healthcare issues, or funding living space or care”). They aspired to create a community living environment in which members could share resources, knowledge and skills so that everyone could invest in their community: “I would like a commune, for all of my sick, queer and disabled friends […] we could all just move in together and help each other”. Sam shared their hopes of living in a polycule (in a family with several partners) so they could support each other and help raise any children they had. They spoke about coming from a large family and how that had benefited their sense of belonging as a child: “The only reason I would maybe even have children of my own is if I had like a big community of people to help”.

Other participants shared their experiences of neglect, abuse, and family estrangement, and how this culminated in unbelonging. Micky reflected on going to a friend’s house for Thanksgiving, as they felt uncomfortable going to their familial home. They had experienced “some abuse and neglect issues”, which meant they no longer spent time with their biological family. Similarly, Ronnie shared that their family were “too indoctrinated into their religion to look past the intolerance it teaches them”. Regrettably, autistic people are at greater risk of experiencing abuse and other traumatic events [77,78], as are trans and gender diverse people [10,11], with the potential of risk of abuse and victimisation higher still for TGD autistic people.

## Discussion

The multi-layered experiences of (un)belonging encountered by TGD autistic participants offer a fascinating example of how different narratives connect and resonate within broader societal and cultural frameworks [38]. Through the narratives presented in this research, we can begin to rethink the concept of (un)belonging in relation to prevailing cis-normative and neuro-normative discourses. For some TGD autistic participants, the experience of unbelonging to cis- and neuro-normative cultures was a considered decision, which included forming chosen families, pursuing self-employment, and joining groups centred around their interests. Participants highlighted the significance of neurodivergent communities and connections, illustrating how the genuine inclusion of TGD autistic individuals in settings such as work, education, and healthcare could enhance their sense of belonging.

### The macro level of (un)belonging

Autistic people have the lowest employment rate amongst disabled people in the UK, with only 22% of autistic adults in employment in 2020 [66]. Similar rates of unemployment affect TGD people in the U.S., who are twice as likely to be unemployed and earn 32% less per year than their cisgender counterparts [79]. In 2023, TGD adults in England and Wales had lower rates of employment (49.2%) and higher rates of unemployment and economic inactivity than their cisgender counterparts (The Equality and Human Rights Monitor). Due to the higher frequency of TGD identities in the autistic community and autism within gender diverse communities [25,80], it is important to understand these specific experiences of (un)belonging at work.

Many of the participants in the current study reported that their workplaces hindered their sense of belonging. A significant number of workplaces fail to modify their environments or expectations for autistic employees due to an insufficient understanding of autism [64]. This, combined with harassment, discrimination, and complicated bureaucratic processes, leaves many autistic individuals unemployed or placed in roles that do not suit their skill sets [64,65]. Half of the study participants chose self-employment to establish safer environments that cater to their needs. Rather than trying to fit into traditional workplaces, many succeeded in creating inclusive workspaces for themselves through self-employment. Notably, participants discussed ableism occurring in their workplaces; however, they did not report any incidents of transphobia in those environments, and the reason for this remains unclear.

### The meso level of (un)belonging

There has been a consistent global increase in autistic people accessing college and university [69], which includes four participants in the current study who were engaged in further education to retrain for work (Anette, Anthony, Micky, Sam). One participant expressed a desire to pursue postgraduate studies to develop skills in community organising and mutual aid, recognising that a sense of belonging involves feeling secure and “at home” [34]. Their efforts included mutual aid for gender-affirming care, in which many participants felt unbelonging in accessing or trying to access. Unbelonging in gender identity healthcare has been extensively documented, indicating that autistic individuals frequently face barriers in obtaining gender identity healthcare because of stigma that presumes they lack awareness, comprehension, and capability [26,42,43]. Unbelonging in gender identity healthcare can result in unmet healthcare needs. Recommendations from this dataset to improve gender identity healthcare have been shared elsewhere [21].

Outside of healthcare, participants shared that they were able to recognise and connect with similar others, with whom they shared identities and cultures. Some participants belonged to autistic groups and spaces that helped create feelings of belonging and community connectedness, which are often absent on a macro level for autistic people [81]. When connecting with similar others, autistic people can discover a sense of belonging, support, and security [71,73,82]. However, one participant (Toby) shared how they felt uncomfortable in *all* social groups, whether autistic or not, due to trauma around their situational muteness (in which a person experiences persistent difficulty in speaking despite their knowledge or comfort with language) [83]. Toby highlights that autistic-only spaces are not a place that *all* autistic people belong, which may suggest another factor in double empathy [19].

Furthermore, participants felt unbelonging in many queer spaces, including experiencing sensory overload in nightclubs, encountering strobe lighting, and the lack of alcohol-free venues and events. Previous work shows that autistic people often leave venues due to sensory and emotional overwhelm [84]. Queer spaces, outside of nightclubbing, were more agreeable. One participant shared how she frequently visited an LGBT+ autistic space, which she found to be “amazing”, whether she attended in person or online. Kim and several other participants felt belonging in virtual spaces, stressing the importance of spatial belonging [32] for TGD autistic people. TGD autistic adults often experience online spaces as fundamental environments for belonging and identity affirmation. Digital platforms can provide accessible avenues for self-expression and mutual support, mitigating exclusion commonly experienced in offline contexts [54]. Online interactions enable TGD autistic adults to explore and validate both gender and neurodivergent identities, fostering a sense of authenticity and shared understanding [85]. Belonging in online contexts is complex – these spaces can offer refuge and empowerment, but they can also be another place where TGD autistic adults encounter exclusion, misunderstanding, or hostility.

The participants’ social connections, both on- and offline, were mostly made through shared identities and experiences (such as LGBT+ groups) as well as hobbies and interests. This indicates that belonging is also based on objects and experiences [31]. Derek, Greg and Anette shared that they would choose their hobbies, interests, and own company over socialising in groups. Belonging to and with objects and experiences (such as going to museums and fossil collecting) was more important for these participants than belonging to social groups. In these instances, we could argue that these TGD autistic people prefer to find belonging in their interests and hobbies, as opposed to with other people.

### The micro level of (un)belonging

Three participants in the current study shared their experiences of living in polycules (a romantic and/or sexual relationship that involves more than two people) [86] or a queer-platonic relationship. For many transgender and gender diverse individuals, the formation of chosen family – networks of friends, partners, and allies who provide mutual care and validation – represents a vital practice of belonging, care, and resistance against heteronormative kinship structures [87]. Such families function as dynamic, intentional communities that reimagine kinship through reciprocity and shared understanding [87]. Chosen families can encompass communal living arrangements, which six participants in the current study reflected would be beneficial for them. These participants indicated that communal living, where members collaborate by sharing resources, knowledge, and skills, could enhance feelings of belonging and connection. Communal living and mutual aid foster a stronger sense of belonging [88] and may particularly benefit TGD autistic individuals who often face increased isolation, abuse, and harm [70,89]. Such networks can mitigate the effects of minority stress, reduce social isolation, and promote resilience and well-being.

Furthermore, digital platforms have increased the opportunities for establishing and maintaining chosen families, enabling geographically dispersed TGD individuals to foster closeness, solidarity, and belonging online [90]. As a result, chosen families not only validate TGD identities but also challenge traditional notions of family and community, promoting flexible, inclusive forms of belonging that emphasise authenticity and self-definition. Ronnie found belonging with other TGD autistic people online due to a lack of support from their biological family – vitally, traditional family structures may not always provide acceptance or affirmation of gender identity [10,11]. Ronnie shared that their family chose religion “over [me]”, which exemplifies the connective nature of macro-level organised religions with micro-level relationships, and how these connections can foster experiences of unbelonging. Similarly, Derek’s micro-level desire to have children was affected by macro-level fertility healthcare policy. Through these stories, we can see that these levels are dynamic and often overlap.

### Strengths and limitations

This study provides a unique contribution to the daily experiences of TGD autistic individuals through the Biographic Narrative Interpretive Method. [37] Understanding (un)belonging across macro, meso, and micro levels allowed us to clarify the relationships between social and political frameworks (including education, employment, healthcare, and education) and collective and personal identities. Employing these levels was both simple yet complex: distinguishing between them posed challenges and differed from person to person, as well as across various personal experiences.

We prioritised depth of understanding over generalisability. Our sample was predominantly White, English-speaking, and college- or university-educated and therefore may not transfer to participants from racialised backgrounds. Regrettably, we could not consider the unique cultural, political, and social contexts that influence various participants since we engaged with individuals from multiple countries. The recruitment strategy influenced the range of perspectives represented. The process may have been inaccessible to those who do not use online spaces, including those who use AAC and do not type, and those with learning disabilities [91–93]. K.M. might have inadvertently selected participants who reflected their own background as a white university graduate. Eleven of the 13 participants were white; one was Chinese, and another was of mixed ethnic background. All participants could complete an interview in English, and 11 of 13 – all but those living in China or France – lived in an English-speaking country: five from the US, 4 from the UK, and one each from Australia and South Africa. Results may not fully be applied in other cultural and linguistic contexts. Future research would be enhanced by a larger participant group from one or two countries (intentionally incorporating those with learning disabilities) to better examine the cultural, political, and social factors that influence TGD autistic people’s experiences of (un)belonging.

Throughout the analysis and presentation of data, we reflected on our experiences – a mix of autistic, non-autistic, cisgender, and transgender. K.M.’s lived experiences were used as a resource for knowledge production to help shape our findings. Their positionality as a trans-masc non-binary autistic person benefited the interview process – two participants commented that they were more comfortable talking to a researcher who shared parts of their identities. The use of our subjective experiences was a crucial part of our reflexive thematic analysis [46,62].

## Conclusion

This study highlights the complex, intersecting experiences of belonging and unbelonging among trans and gender diverse (TGD) autistic individuals, examined across macro, meso, and micro levels. At the macro level, participants faced significant barriers to employment and inclusion, reflecting broader issues of systemic discrimination in both disability and gender contexts. Many participants reported that traditional workplaces were inaccessible or unaccommodating, leading many to pursue self-employment as a means of creating safer, more inclusive environments. At the meso level, participants’ experiences in education and healthcare highlighted persistent gatekeeping and stigma, particularly in accessing autism diagnoses and gender-affirming care. While some found belonging in autistic or LGBT+ communities, others experienced sensory or social difficulties that limited participation, suggesting the need for more accessible and intersectional queer spaces. Online communities and shared interests similarly appeared as important sources of belonging. At the micro level, chosen families and alternative kinship structures were central to participants’ sense of belonging. This study indicates that communal living and mutual aid could enhance connectedness and mitigate isolation for TGD autistic individuals, demonstrating that belonging is shaped through dynamic interactions between structural conditions, community spaces, and interpersonal relationships.

## Supporting information

S1 File COREQ Checklist. (PDF)
